# Approaches for modelling autocorrelation function and data processing in time-domain diffuse correlation spectroscopy

**DOI:** 10.1364/BOE.577785

**Published:** 2025-12-01

**Authors:** Aleh Sudakou, Ilias Tachtsidis, Michal Kacprzak, Adam Liebert, Stanislaw Wojtkiewicz

**Affiliations:** 1 Nalecz Institute of Biocybernetics and Biomedical Engineering, Polish Academy of Sciences, Warsaw, Poland; 2 Department of Medical Physics and Biomedical Engineering, University College London, London, UK

## Abstract

Time-domain diffuse correlation spectroscopy (TD-DCS) is a non-invasive optical technique for measuring tissue blood flow. Recovering the blood flow index (*αD*_b_) requires accurate modelling of the normalised electric field autocorrelation function (*g*_1_), and an optimised data processing approach to minimise noise. We quantitatively compared four modelling approaches for *g*_1_: (i) using momentum transfer (*Y*) and pathlengths (*L*) from Monte Carlo (MC) simulations, (ii) using *L* only, (iii) applying an analytical solution of the photon diffusion equation (DE) in time domain, and (iv) applying an analytical solution of the correlation diffusion equation (CDE) in steady state. The second and third approaches use solutions in near-infrared spectroscopy (NIRS) for modelling *g*_1_ in DCS by assuming *Y* = *μ′*_s_*L*. We computed *g*_1_ curves using the first approach, considered the gold standard, and recovered *αD*_b_ using the other three approaches for various source-detector distances (*ρ*) and scattering coefficients (*μ′*_s_). Also, we investigated how the correlator time bin width (*T*_bin_), which is an adjustable parameter in data processing, affects the standard deviation of *g*_1_ (or the normalised intensity autocorrelation function *g*_2_). We used a more convenient version of the noise equation expressed as a function of *g*_1_ (or *g*_2_), which removes the need to know the decay rate. When using photons detected after ∼0.5 ns, all four approaches produced nearly identical *g*_1_ curves. Using all detected photons, the DE solution produced negligible errors (up to ∼2%) in the recovered *αD*_b_ across various *ρ* and *μ′*_s_, while using *L* from MC simulations resulted in larger errors (up to ∼9% at *ρ* = 5 mm and ∼1.5% at *ρ* = 30 mm). The analysis of the probability distributions *P*(*Y*) and *P*(*μ′*_s_*L*) explained these differences. As expected, the standard deviation of *g*_1_ (or *g*_2_) can be reduced during data processing by increasing *T*_bin_. To achieve the lowest standard deviation, *T*_bin_ should be longer than the inverse of the photon count rate, indicating that the optimal *T*_bin_ may vary across different time gates. The results provide quantitative insights into modelling *g*_1_ (or *g*_2_), and provide a direct guideline for minimising the standard deviation of *g*_1_ (or *g*_2_) in data processing.

## 1. Introduction

Monitoring cerebral blood flow (CBF) dynamics provides vital information about brain health and function. It can aid in diagnosing conditions such as stroke, traumatic brain injury, neurodegenerative diseases, and others, allowing timely and effective treatment [1]. Diffuse correlation spectroscopy (DCS) is a non-invasive optical technique that measures microvascular blood flow in tissues, providing a quantitative absolute blood flow index (BFI in cm^2^s^-1^), which is directly related to CBF [2,3]. A variety of in vivo validation studies have confirmed that DCS measurements correlate with the average blood flow inside tissue microvasculature, as compared to other techniques [4]. The current status of DCS technology and future outlook were recently reviewed in [5], and in [6] with an emphasis on the human brain, including the impact on neuroscience and clinical applications. The technological advances were also reviewed in [7]. Other optical methods, such as Laser Speckle Contrast Imaging (LSCI) and Diffusing Wave Spectroscopy (DWS), also measure blood perfusion, but they collect and analyse the light signal differently, examining different aspects of the same temporal autocorrelation function [8–10]. Microcirculation imaging techniques were reviewed in [11].

The signal in DCS is the temporal autocorrelation function of the detected light intensity $\left(g_{2}\right)$ . The decay rate of $g_{2}$ is proportional to the movement of scatterers, which are the red blood cells (RBCs) inside tissue’s microvasculature [12]. Advances in lasers and detectors [2,7] have allowed the more challenging time-domain DCS (TD-DCS) technique [13,14]. TD-DCS enables pathlength-resolved analysis of $g_{2}$ using different time gates, allowing separation of signal contributions from shallow and deep layers (scalp and brain in measurements on the head), providing potentially more accurate assessment of CBF. Milej et al. [15] demonstrated the extracerebral signal contamination on optical measurements of CBF, which is a well-known major challenge in DCS and NIRS [16].

Modelling $g_{2}$ is essential in interpreting measurements and recovery of BFI [3,17,18], optimising measurement systems’ parameters [19,20], and testing data analysis methods [21]. The radiative transfer equation (RTE) accurately describes the propagation of light through a medium, and the most commonly employed solution in near-infrared spectroscopy (NIRS) is the photon diffusion equation (DE), which relies on the diffusion approximation [22,23]. The DE models the diffusion of photons resulting from scattering and propagation, and includes a loss term due to absorption. The signal in time-domain NIRS (TD-NIRS) is the distribution of times of flight of photons (DTOFs), which allows assessment of tissue optical properties, i.e., the absorption (*μ*_a_) and reduced scattering (*μ′*_s_) coefficients [24]. The correlation diffusion equation (CDE) is a modified form of the DE, in which *μ*_a_ is replaced with a dynamic absorption term *μ*_d_(*τ*) that increases with correlation time *τ* and accounts for the loss of coherence caused by scattering from moving particles, as measured in DCS [3,17,18]. Analytical solutions of the DE can be directly adopted for solving the CDE by replacing *μ*_a_ with *μ*_d_(*τ*). Boas et al. [12,17] derived a method for modelling the temporal electric field autocorrelation function $\left(g_{1}\right)$ using Monte Carlo (MC) simulations, which is considered the gold standard approach for modelling $g_{1}$ . For each detected photon packet, the pathlength (*L*) and momentum transfer (*Y*) are accumulated over all scattering events, which allows calculation of $g_{1}$ . The method based on *L* and *μ*_d_(*τ*) will yield identical $g_{1}$ as the method based on *Y* when *Y* = *μ′*_s_*L*.

The first aim of this study was to quantitatively compare different approaches for modelling $g_{1}$ , using the first approach based on *Y* from MC simulations [12,17] as a reference. The second approach uses *L* from MC simulations and *μ*_d_(*τ*), as in [25]. The third approach uses an analytical solution of the DE in time domain and *μ*_d_(*τ*), as in [21]. The fourth approach uses an analytical solution of the CDE for a semi-infinite homogeneous medium in steady state [17,18], which is the most commonly used method for analysing DCS measurements. We quantitatively compare *Y* and *μ′*_s_L, as well as their probability distributions *P*(*Y*) and *P*(*μ′*_s_*L*), to determine the source of the discrepancies between different approaches. The distribution of pathlengths *P*(*L*) is the normalised DTOF. Therefore, analytical solutions of the DE can be used to obtain *P*(*μ′*_s_*L*).

The second aim of the study addresses the data processing in DCS using the modelling of noise. The commonly used equation for the standard deviation of $g_{1}(\sigma_{g_{1}})$ in DCS was presented by Zhou et al. [26,27], which is based on the equation derived by Koppel [28]. We propose expressing the equation for $\sigma_{g_{1}}$ directly as a function of $g_{1}$ , as was similarly done in fluorescence correlation spectroscopy by Wohland et al. [29]. TD-DCS uses a software correlator [30] for computing $g_{2}$ , as opposed to a hardware correlator which is typically used in CW-DCS. An advantage of a software correlator is the flexibility in choosing its settings, which are typically hard-wired in hardware correlators. We assessed the effect of data processing parameters, e.g., the time bin width (*T*_bin_), on the calculated $\sigma_{g_{1}}$ , which is proportional to the standard deviation of the recovered BFI.

## 2. Methods

### 2.1. Approaches for modelling $g_{1}$

Here, we describe four approaches for modelling the normalised autocorrelation function of the electric field $g_{1}(\tau ,t_{\text{a}})$ , where τ is the delay time and $t_{\text{a}}$ is the selected range of photon arrival times. $t_{\text{a}}$ determines which photons are used in the computation of $g_{1}$ . Equivalently, photons can be selected based on a range of pathlengths *L*, for computing pathlength-resolved $g_{1}(\tau ,L)$ .

The Monte Carlo (MC) code [31], described in Section 2.3, records the total pathlength (*L_i_*) and momentum transfer (*Y_i_*) in each tissue type or layer (*i*), as well as the total number of scattering events, for each detected photon packet. In the case of Brownian motion, where scatterers move in random directions, the momentum transfer for a single scattering event can be calculated as 1 – cos(*θ*) [17], where *θ* is the scattering angle. The probability distribution function of *θ* can be expressed with the modified Henyey-Greenstein phase function [32,33].

**The first approach** uses *L_i_* and *Y_i_* generated by MC simulations [12,17], which is considered the gold standard approach. The non-normalised autocorrelation function of the electric field $G_{1}(\tau ,t_{\text{a}})$ can be calculated as:

(1) $$G_{1}(\tau ,t_{\text{a}})=\frac{1}{N_{\text{tot}}}\overset{N_{\text{tot}}}{\underset{p=1}{\sum }}\text{exp}\left(-\frac{1}{3}\overset{N_{\text{tiss}}}{\underset{i=1}{\sum }}k_{0,i}^{2}\;Y_{i,p}\;\left\langle \Delta r_{i}{\left(\tau \right)}^{2}\right\rangle \right)\text{exp}\left(-\overset{N_{\text{tiss}}}{\underset{i=1}{\sum }}\mu_{\text{a},i}\;L_{i,p}\right)$$

where *N*_tot_ is the total number of detected photon packets, *N*_tiss_ is the number of tissue types or layers indexed by *i*, *k*_0,*i*_ is the wavelength number of light, defined as *k*_0,*i*_ = 2π*n_i_* / λ, where λ is the wavelength and *n_i_* is the refractive index. *μ*_a,*i*_ is the absorption coefficient. $\left\langle \Delta r_{i}{\left(\tau \right)}^{2}\right\rangle$ is the mean square displacement of the scattering particles after time *τ*.

We present all expressions in terms of $\left\langle \Delta r_{i}{\left(\tau \right)}^{2}\right\rangle$ , and in all data analyses we substituted the relation given in Eq. (2). It was found that the decay rate of $g_{2}$ is proportional to the delay time (τ), rather than τ^2^, establishing that the DCS signal is better described by Brownian diffusion model of the red blood cells rather than a convective flow model [12]. Mixed models can better fit the measured data [34–37], but Brownian diffusion remains the most used flow model, for which:

(2) $$\left\langle \Delta r_{i}{\left(\tau \right)}^{2}\right\rangle =6D_{\text{b},i}\tau$$

where *D*_b_ is the Brownian diffusion coefficient. The product *αD*_b_ is commonly referred to as the blood flow index (BFI) [37]. *α* represents the probability of light scattering by moving scatterer, corresponding to the fraction of moving particles within a tissue. In this study, we set *α* = 1, which is a scaling factor and does not affect any of the findings. In parameter recovery, when both *α* and *D*_b_ are unknown, we can recover only their product *αD*_b_.

**The second approach** uses *L_i_* only [13,17], as in [25]. As explained in the Introduction, solutions for the distribution of times of flight of photons (DTOFs) in NIRS can be used to obtain solutions for pathlength-resolved $g_{1}$ by replacing *μ*_a_ with the dynamic term *μ*_d_(*τ*), which increases with τ and accounts for the loss of coherence. The DTOF can be calculated as:

(3) $$\text{DTOF}(t_{\text{a}})=\overset{N_{\text{tot}}}{\underset{p=1}{\sum }}\text{exp}\left(-\overset{N_{\text{tiss}}}{\underset{i=1}{\sum }}\mu_{\text{a},i}L_{i,p}\right)$$

Replacing *μ*_a_ with $\mu_{\text{d}}(\tau )=\mu_{\text{a}}+1/3\mu_{\text{s}}^{\prime }\;k_{0}^{2}\;\left\langle \Delta r{\left(\tau \right)}^{2}\right\rangle$ [17] results in the following expression, which is identical to Eq. (1) when *Y* = *μ′*_s_*L*:

(4) $$G_{1}(\tau ,t_{\text{a}})=\text{DTOF}(\tau ,t_{\text{a}})=\overset{N_{\text{tot}}}{\underset{p=1}{\sum }}\text{exp}\left(-\frac{1}{3}\overset{N_{\text{tiss}}}{\underset{i=1}{\sum }}k_{0,i}^{2}\;\mu_{\text{s},i}^{\prime }\;L_{i,p}\;\left\langle \Delta r_{i}{\left(\tau \right)}^{2}\right\rangle \right)\text{exp}\left(-\overset{N_{\text{tiss}}}{\underset{i=1}{\sum }}\mu_{\text{a},i}L_{i,p}\right)$$

**The third approach** applies an analytical solution of the DE in time domain, with *μ*_a_ replaced by *μ*_d_(*τ*). We used the DE solution for an N-layered medium presented by Liemert et al. [38], which uses the Laplace transform instead of the commonly used Fourier transform [39]. The solution was previously implemented in MATLAB and made publicly available [40].

**The fourth approach** applies an analytical solution of the CDE in steady state for a homogeneous medium [17,18], which is the most commonly used approach for analysing CW-DCS data:

(5) $$G_{1}(\tau )=\frac{3\mu_{\text{s}}^{\prime }}{4\pi }\left[\frac{\text{exp}(-K(\tau )\;r_{1})}{r_{1}}-\frac{\text{exp}(-K(\tau )\;r_{2})}{r_{2}}\right]$$

where $K{\left(\tau \right)}^{2}=3\mu_{s}^{\prime }\mu_{d}(\tau ),\;r_{1}^{2}=\rho^{2}+z_{0}^{2},\;r_{2}^{2}=\rho^{2}+{\left(z_{0}+2z_{\text{b}}\right)}^{2},\;z_{0}=\mu_{\text{s}}^{\prime -1}$ , and $z_{\text{b}}=2(1+R_{\text{eff}})/(1-R_{\text{eff}}){\left(3\mu_{\text{s}}^{\prime }\right)}^{-1}$ . The variable *ρ* is the source-detector distance, and *R*_eff_ is the internal reflection coefficient. *R*_eff_ accounts for the refractive index mismatch between the medium and the outside, and is related to the assumed boundary conditions [23]. In contrast to the first three approaches, Eq. (5) applies only to CW-DCS.

When *τ* = 0, *μ*_d_(*τ*) simplifies to *μ*_a_ and Eq. (5) simplifies to the DE solution [41]. Similarly, Eqs. (1), (3), and (4) are identical at *τ* = 0. $g_{1}$ can be calculated by dividing *G*_1_ by its value at *τ* = 0:

(6) $$g_{1}(\tau ,t_{\text{a}})=\frac{G_{1}(\tau ,t_{\text{a}})}{G_{1}(\tau =0,t_{\text{a}})}\;\text{and}\;g_{1}(\tau ,t_{\text{a}})=\frac{\text{DTOF}(\tau ,t_{\text{a}})}{\text{DTOF}(\tau =0,t_{\text{a}})}$$

For a homogeneous medium, the weights exp(–*μ*_a_ *L*) of photon packets can be accumulated into a histogram to obtain the probability distribution of momentum transfer *P*(*Y*) [17] or of pathlength *P*(*μ′*_s_*L*), depending on whether the weights are binned by *Y* or by *μ′*_s_*L*. The histograms are normalised by their integral. *P*(*μ′*_s_*L*) can be obtained using the DE solution by first calculating the normalised DTOF $\left(t_{\text{a}}\right)$ and then converting $t_{\text{a}}$ to *μ′*_s_*L* using *L* *=* *ct_a_*, where *c* is the speed of light in the medium. The correlation function $g_{1}$ can be calculated as:

(7) $$g_{1}(\tau ,t_{\text{a}})=\overset{Y_{\text{max}}}{\underset{Y=0}{\sum }}P(Y)\;\text{exp}\left(-\frac{1}{3}k_{0}^{2}\;Y\;\left\langle \Delta r{\left(\tau \right)}^{2}\right\rangle \right)$$

(8) $$g_{1}(\tau ,t_{\text{a}})=\overset{L_{\text{max}}}{\underset{L=L_{\text{min}}}{\sum }}P(\mu_{\text{s}}^{\prime }\;L)\;\text{exp}\left(-\frac{1}{3}k_{0}^{2}\;\mu_{\text{s}}^{\prime }\;L\;\left\langle \Delta \text{r}{\left(\tau \right)}^{2}\right\rangle \right)$$

Equations (7) and (8) result in identical $g_{1}$ curves as Eq. (1) and (4), respectively, after normalisation.

### 2.2. Noise model

Koppel [28] presented a method for deriving an equation for the standard deviation of a general correlation function, and derived it for an exponentially decaying correlation function. Koppel described how the method applies to fluorescence correlation spectroscopy (FCS), as well as to laser light-scattering experiments such as in DCS. Zhou et al. [26,27] applied the equation derived by Koppel [28] to DCS measurements, which became the most commonly used method for calculating the theoretical standard deviation of $g_{1}$ and $g_{2}$ in DCS, requiring knowledge of the decay rate of $g_{1}$ or $g_{2}$ .

Wohland et al. [29] proposed expressing the equation for the standard deviation of $g_{1}(\sigma_{g_{1}})$ in FCS as a function of $g_{1}$ rather than the decay rate. The authors showed that the equation approximates $\sigma_{g_{1}}$ for the measured intensity fluctuations. Here, we similarly express $\sigma_{g_{1}}$ (or $\sigma_{g_{2}}$ ) in DCS as a function of $g_{1}$ (or $g_{2}$ ) rather than the decay rate. The derivation of Eqs. (9) and (10) are briefly explained in the Appendix. The equation for $\sigma_{g_{2}}$ is:

(9) $$\begin{array}{ll} \sigma_{g_{2}}{\left(\tau \right)}^{2} & =\frac{T_{\text{bin}}}{T_{\text{meas}}}\frac{\left(\beta^{2}+{\left(g_{2}(T_{\text{bin}})-1\right)}^{2})(\beta^{2}+{\left(g_{2}(\tau )-1\right)}^{2}\right)}{\beta^{2}-{\left(g_{2}(T_{\text{bin}})-1\right)}^{2}}+\frac{2\tau \;{\left(g_{2}(\tau )-1\right)}^{2}}{T_{\text{meas}}}\\ & +\frac{2(\beta +{\left(g_{2}(\tau )-1\right)}^{2}/\beta )}{N_{\text{tot}}}+\frac{T_{\text{meas}}\;g_{2}(\tau )}{T_{\text{bin}}\;{N_{\text{tot}}}^{2}} \end{array}$$

where *T*_bin_ is the time bin, which is the time over which the detected photons are accumulated. It determines the resolution in *τ*, so it should be equal or greater than the smallest *τ* at which the $g_{1}$ begins to decay. *T*_bin_ is adjustable in software correlators, such as in TD-DCS, but typically fixed in hardware correlators in CW-DCS. *T*_meas_ is the total measurement time. *β* is the coherence factor that depends on the properties of the experimental setup, and for an ideal system *β* = 1 [13]. For TD-DCS, the value of *β* depends also on the time gate [13,42]. The equation for $\sigma_{g_{1}}$ is:

(10) $$\begin{array}{l} \sigma_{g_{1}}{\left(\tau \right)}^{2}=\frac{T_{\text{bin}}}{T_{\text{meas}}}\frac{\left(1+g_{1}{\left(T_{\text{bin}}\right)}^{2})(1+g_{1}{\left(\tau \right)}^{2}\right)}{1-g_{1}{\left(T_{\text{bin}}\right)}^{2}}+\frac{2\tau g_{1}{\left(\tau \right)}^{2}}{T_{\text{meas}}}+\frac{2(1+g_{1}{\left(\tau \right)}^{2})}{N_{\text{tot}}}\\ \;+\frac{T_{\text{meas}}}{T_{\text{bin}}}\frac{\left(1+g_{1}(\tau )\right)}{{N_{\text{tot}}}^{2}} \end{array}$$

The first two terms in Eqs. (9) and (10) do not depend on *N*_tot_. Therefore, the uncertainty will not improve indefinitely as the count rate increases, which was discussed by Koppel [28]. The second term is inversely proportional to *T*_meas_, as expected for the uncertainty in repeated or prolonged measurements.

*T*_bin_ and *τ* must be much smaller than the time scatterers require to move a distance equal to the wavelength of light. For typical DCS measurements on tissues characterised by *D*_b_ ≈ 10^6^ mm^2^s^−1^, this requires that *τ* ≪ 10^−3^ s [17]. Additionally, Koppel’s equation was derived under the assumption of a high number of moving particles within the volume penetrated by photons, negligible background light, and uniform illumination [29, 43].

### 2.3. Monte Carlo simulations

The massive-parallel, multi-purpose MC code for simulation of light propagation through complex structures was previously developed and described by Wojtkiewicz and Liebert [31]. For this study, we have added the option to output the total pathlength (*L_i_*), the total momentum transfer (*Y_i_*), and the number of scattering events for each tissue type or layer (*i*), for each detected photon packet. MC outputs were saved to a text file, and data analysis was carried out in MATLAB. We used the ‘zero boundary condition’ [23,44]. In MC simulations, the boundary condition was implemented by terminating all photons that encountered the outside boundary. For analytical solutions, we set *n*_outside_ = *n*_medium_, which results in no internal reflections (*R*_eff_ = 0).

**Fig. 1.**
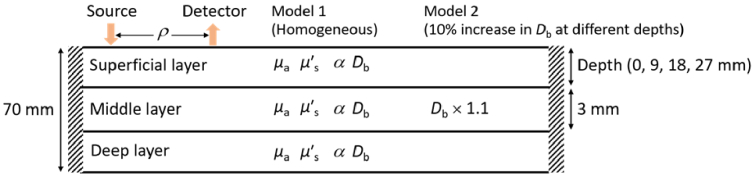
Schematic of the simulated models: a homogeneous medium (Model 1) and a medium with a 10% increase in *D*_b_ within a 3 mm layer at different depths (Model 2).

We used either a homogeneous model or a three-layered model, as shown in Fig. 1. Depth-dependent contrast was simulated by increasing *D*_b_ by 10% within a 3 mm layer starting at 0, 9, 18, or 27 mm depth. Unless otherwise stated, the optical properties were set to those of typical adult head tissue [45]: *μ*_a_ = 0.01 mm^-1^, *μ′*_s_ = 1 mm^−1^, *g* = 0.9, and *n* = 1.4. The comparison between *μ*′_s_*L* and *Y* was repeated for *g* = 0, and the results are presented in the Appendix. *ρ* was varied from 5 to 30 mm.

The model dimensions were 200 mm by 200 mm by 70 mm (depth), with a voxel size of 0.25 mm. The source and detectors were positioned on the surface. The source (diameter 0.5 mm) was placed at the centre, and circular detectors were placed around it at distance *ρ*, as illustrated in [46]. The detectors diameter increased from 0.25 mm at *ρ* = 5 mm to 2.5 mm at *ρ* = 30 mm to collect a sufficient number of photon packets for statistical analysis. For the same detection area, *N*_tot_ decreases roughly one order of magnitude for a 10 mm increase in *ρ*. The typical diameter of a detection fibre in TD-NIRS measurements is ∼3 mm [47]. We launched 10^9^ photon packets at *ρ* = 5 mm and up to 5 × 10^9^ at *ρ* = 30 mm, resulting in *N*_tot_ ranging from 4 × 10^6^ to 19 × 10^6^. A larger *N*_tot_ was necessary at *ρ* = 30 mm to obtain a sufficient number of photon packets at the earliest arrival times.

## 3. Results

### 3.1. Comparison of approaches for modelling $g_{1}$

Here, we present a comparison of *μ*′_s_*L* and *Y* for forward scattering (*g* = 0.9 and *μ*_s_ = 10 mm^−1^), and the results for isotropic scattering (*g* = 0 and *μ*_s_ = 1 mm^−1^) are presented in the Appendix. By definition, *μ*′_s_ = (1 – *g*) *μ*_s_. We set *μ*′_s_ = 1 mm^-1^, such that the numerical value of *μ*′_s_*L* (unitless) is the same as that of *L* (mm).

**Fig. 2.**
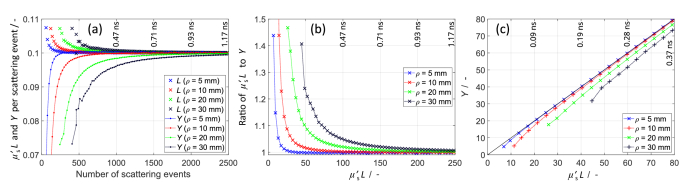
Comparison of pathlength (*μ*′_s_*L*) and momentum transfer (*Y*). (a) Mean *μ*′_s_*L* and *Y* per scattering event after different number of scattering events. (b) Ratio of *μ*′_s_*L* to *Y* at different photon arrival times $(t_{\text{a}})$ , where $t_{\text{a}}$ is converted to *L* using *L* = *ct_a_*. (c) Comparison of *μ*′_s_*L* and *Y*. The corresponding $t_{\text{a}}$ values (units ns) are indicated in each panel.

Figure 2(a) shows *μ*′_s_*L* and *Y* per scattering event, averaged over all detected photon packets. Fully diffused photon packets (many scattering events) on average travelled 0.1 mm and experienced 0.1 momentum transfer per scattering event, as expected and shown in Fig. 2(a). Photon packets that travelled short pathlengths (few scattering events) had smaller than average scattering angles (resulting in less than 0.1 *Y* per scattering), consistent with [17], and longer than average step lengths (greater than 0.1 mm *L* per scattering). For larger *ρ*, photons require more scattering events to become fully diffuse.

We divided the arrival time of photons into 0.018 ns time gates and calculated the mean *μ*′_s_*L* $\left(t_{\text{a}}\right)$ and *Y* $(t_{\text{a}})$ of detected photon packets within each time gate $t_{\text{a}}$ . Figure 2(b) shows the ratio of *μ*′_s_*L*(*t_a_*) to *Y*(*t_a_*), where $t_{\text{a}}$ was converted to *L* on the x-axis using *L* = *ct_a_*. Photons must travel more than ∼25 mm or ∼200 mm to be nearly fully diffused at *ρ* = 5 mm or *ρ* = 30 mm, respectively (Fig. 2(b)). *μ*′_s_*L* is always greater than *Y*. Therefore, to produce a similar $g_{1}$ curve, Eq. (1), which uses *Y*, should use a larger *αD*_b_ value than Eq. (4), which uses *μ*′_s_*L*. If $g_{1}$ is obtained using Eq. (1) and *αD*_b_ is recovered using Eq. (4), it will always be underestimated. Figure 2 does not include information about *αD*_b_, so the findings apply for any *αD*_b_ value.

Figure 2(c) shows *Y* versus *μ*′_s_*L*. The shortest *L* is ∼12 mm at *ρ* = 10 mm and ∼45 mm at *ρ* = 30 mm. As *ρ* increases, the probability that photons travel almost straight to a detector (without significant scattering) decreases.

**Fig. 3.**
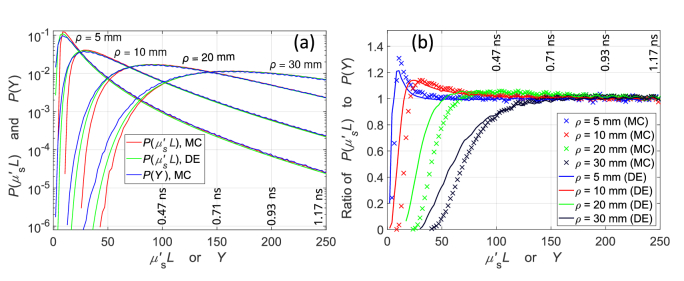
(a) Comparison of the probability distributions of pathlength *P*(*μ*′_s_*L*) and momentum transfer *P*(*Y*) calculated using *L* and *Y* from MC simulations or an analytical solution of the DE. (b) Ratio of *P*(*μ*′_s_*L*) to *P*(*Y*). The x-axis represents *μ*′_s_*L* for *P*(*μ*′_s_*L*) and *Y* for *P*(*Y*).

Figure 2 shows that for longer *ρ*, there is a worse match between *μ*′_s_*L* and *Y* at short *L*. However, Fig. 2 lacks information about the number of detected photons, and there are far fewer early photons for longer *ρ*. Figure 3(a) presents the probability distributions *P*(*Y*) and *P*(*μ′*_s_*L*) calculated using the same MC data as in Fig. 2, along with *P*(*μ′*_s_*L*) calculated using the time-domain DE solution. The DE solution produces *P*(*μ′*_s_*L*) that is in-between *P*(*Y*) and *P*(*μ′*_s_*L*) calculated using MC data. This is consistent with the results in Fig. 2(a), where *μ′*_s_*L* is higher than 0.1 and *Y* is lower than 0.1, whereas the DE solution would have a constant *μ′*_s_*L*, (0.1) regardless of the number of scattering events.

Figure 3(b) presents the ratios of *P*(*μ′*_s_*L*) to *P*(*Y*), highlighting their differences. The ratios start at 0 for short *L*, consistent with the results in Fig. 2(c), where *Y* is smaller than the smallest *μ′*_s_*L* values. Figure 2(b) shows the magnitude of the discrepancy between *μ′*_s_*L* and *Y* at different *L*, whereas Fig. 3(a) shows how many photons are detected with those discrepancies. The discrepancy at early $t_{\text{a}}$ increases with *ρ* (Fig. 2(b)), but the amount of detected early photons greatly decreases (Fig. 3(b)). At *ρ* = 30 mm, most photons travel ∼150 mm or more inside the medium before reaching a detector (Fig. 3(a)).

**Fig. 4.**
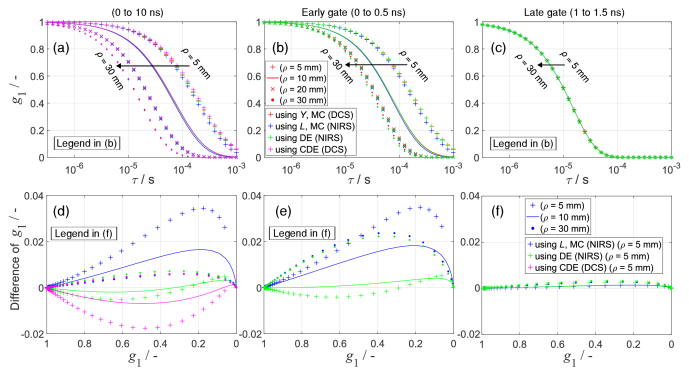
Comparison of $g_{1}$ curves computed using four modelling approaches for three time gates: (a, d) all photons as in CW-DCS, (b, e) early gate, and (c, f) late gate. Top row: $g_{1}$ curves at four *ρ* values. Bottom row: differences in $g_{1}$ curves relative to the approach based on *Y* from MC.

Figure 4 (top row) compares $g_{1}$ curves computed using four different approaches, for four values of *ρ*. Using photons with all pathlengths, as in CW-NIRS, the decay rate of $g_{1}$ increases with *ρ* (Fig. 4(a)). Using photons with arrival times after 1 ns, the $g_{1}$ curves are independent of *ρ* (Fig. 4(c)). Increasing the gate time increases the decay rate (Fig. 4(b, c)). All approaches produced similar $g_{1}$ curves.

Figure 4 (bottom row) shows the discrepancies in $g_{1}$ values computed using different approaches compared to the most accurate first approach (using *Y*). The differences are plotted relative to the $g_{1}$ values to show the magnitude of the discrepancies. In typical DCS data analysis, $g_{1}$ values close to 0 are not used. Notably, the discrepancies are smaller when using the DE solution (third approach) than when using *L* from MC simulations (second approach).

**Fig. 5.**
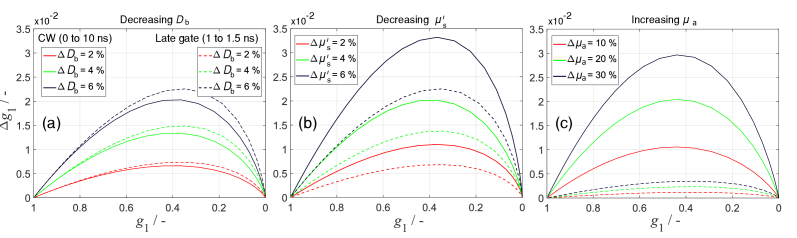
Changes in $g_{1}$ curves resulting from percentage changes in (a) *D*_b_, (b) *μ′*_s_, and (c) *μ*_a_. Results are shown for all photons (0 to 10 ns, solid lines) and for the late gate (1 to 1.5 ns, dashed lines). The resulting curves are comparable to those in the bottom row of Fig. 4.

Figure 5 shows changes in *g*_1_ (Δ*g*_1_) resulting from percent changes in *D*_b_, *μ*′_s_, or *μ*_a_. The first modelling approach (using *Y*) was used with *ρ* = 20 mm. Results for the early gate (not shown) were between those for CW-DCS (0 to 10 ns) and the late gate (1 to 1.5 ns), being much closer to the CW-DCS results. The shapes of Δ*g*_1_ curves in Fig. 5(a) are similar to those in Fig. 4(d, e) for *ρ* = 30 mm. Therefore, different modelling approaches can produce nearly the same $g_{1}$ curves but with different *D*_b_ values. Δ*g*_1_ is linearly proportional to percent changes in *D*_b_, *μ*′_s_, and *μ*_a_. Changes in *μ*_a_ have almost no effect on $g_{1}$ when using photons with arrival times after 1 ns.

**Fig. 6.**
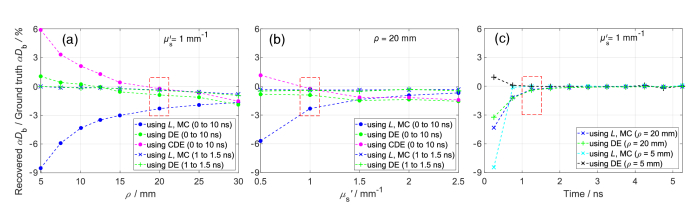
Percentage errors in recovered *αD*_b_ using different modelling approaches within the fitting-based recovery process, for varying *ρ* (a), *μ*′_s_ (b), and the position of a 0.5 ns time gate (c). The $g_{1}$ values for the ground truth *αD*_b_ were computed using the modelling approach based on *Y* from MC simulations. Red dashed squares indicate the same cases across panels.

We computed $g_{1}$ curves using the first modelling approach (using *Y*) for various *ρ*, *μ*′_s_, and time gates, and then recovered *αD*_b_ using the other modelling approaches and the Levenberg–Marquardt algorithm (LMA). Figure 6 shows the percent errors in recovered *αD*_b_. The results are independent of the chosen ground truth *αD*_b_ since errors are reported in percentages, consistent with Fig. 5(a) where Δ*g*_1_ is proportional to percentage change in *D*_b_. The largest errors occur when the diffusion approximation is not fully satisfied, i.e., for short *ρ* (Fig. 6(a)), small *μ*′_s_ (Fig. 6(b)), and early photons (Fig. 6(c)).

*αD*_b_ recovered using the second approach (using *L* only) is always underestimated, as expected from Fig. 2(b), where *μ*′_s_*L* is always larger than *Y*. The third approach (using the DE solution) accurately recovers *αD*_b_ across all *ρ*, *μ*′_s_, and time gates, with the largest error of ∼3% (Fig. 6(c)).

### 3.2. Standard deviation of $g_{1}$ : optimising T_bin_, τ, and time gate

**Fig. 7.**
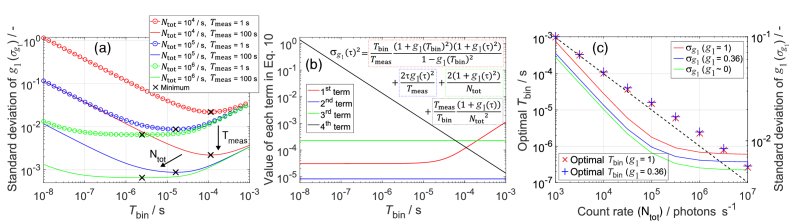
Optimisation of the correlator time bin width (*T*_bin_). (a) Standard deviation of $g_{1}(\sigma_{g_{1}})$ versus *T*_bin_ for different count rates (*N*_tot_) and measurement times (*T*_meas_). Black crosses indicate the minima. (b) Values of each term in Eq. (10). (c) Optimal *T*_bin_, which minimises $\sigma_{g_{1}}$ , versus *N*_tot_ for three $g_{1}$ values; markers correspond to the left y-axis and lines to the right y-axis.

**Fig. 8.**
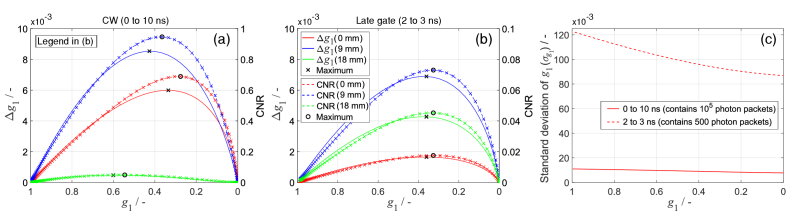
Optimisation of the range of $g_{1}$ values for assessing *D*_b_ changes at different depths. Contrast (Δ*g*_1_) and CNR resulting from a 10% increase in *D*_b_ at different depths using (a) using all photons, and (b) using a late time gate. (c) Standard deviation of $g_{1}$ used to calculate CNR. The CNR axis scale in (b) is 10 times smaller than in (a).

**Fig. 9.**
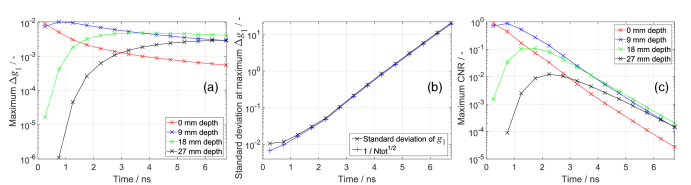
Optimisation of the 0.5 ns time gate position for assessing *D*_b_ changes at different depths. Contrast was induced by a 10% increase in *D*_b_ within a 3 mm layer at different depths, as indicated in the legend. (a) Maximum Δ*g*_1_ at different photon arrival times, (b) standard deviation of the corresponding *g*_1_, and (c) maximum CNR.

We have analysed how the theoretical standard deviation of $g_{1}(\sigma_{g_{1}})$ , given by Eq. (10), depends on *T*_bin_ and count rate *N*_tot_ (Fig. 7), the value of $g_{1}$ (Fig. 8), and the time gate position (Fig. 9). Equation (10) is provided in Fig. 7(b). We used the $g_{1}$ curve for *ρ* = 20 mm shown in Fig. 4(a) to calculate $g_{1}(T_{\text{bin}})$ and $g_{1}(\tau )$ in Eq. (10).

Figure 7(a) shows $\sigma_{g_{1}}$ at *τ* = 3.2 × 10^−5^ s, where $g_{1}$ is approximately 0.36, as a function of *T*_bin_ for two values of *T*_meas_ (1 and 100 s) and three values of *N*_tot_ (10^4^, 10^5^, and 10^6^ photons s^−1^). Increasing *T*_meas_ reduces $\sigma_{g_{1}}$ by a factor of $\sqrt{T_{\text{meas}}}$ , analogous to averaging over repeated measurements. Similarly, for small *T*_bin_ (∼10^−8^ s), increasing *N*_tot_ reduces $\sigma_{g_{1}}$ by a factor of $\sqrt{N_{\text{tot}}}$ . In contrast, when *T*_bin_ is long (∼10^−3^ s), *N*_tot_ has a negligible effect on $\sigma_{g_{1}}$ . Note that when using long *T*_bin_ (e.g., ∼10^4^ s), the shortest *τ* (i.e., ∼10^−4^ s) is near the end of the decay region of $g_{1}$ (see Fig. 4). Therefore, the calculated $\sigma_{g_{1}}$ for *T*_bin_ longer than ∼5 × 10^−5^ s are invalid because $g_{1}$ curve becomes a constant number, i.e., 0, highlighting the limitation of the noise model and that it is meaningless to calculate $\sigma_{g_{1}}$ after $g_{1}$ has decayed to 0. $\sigma_{g_{1}}$ decreases until *T*_bin_ ≈ 2 *N*_tot_^−1^, when the average number of photons per *T*_bin_ ( ⟨n⟩ in the Appendix) is a few photons.

Figure 7(b) shows the values of the four terms in Eq. (10) as a function of *T*_bin_. These values strongly depend on the chosen parameters (*N*_tot_ = 10^4^, *T*_meas_ = 1 s, and *g*_1_ = 0.36), consistent with Fig. 7(c). The first term is constant until $g_{1}$ decays to 0, after which it is not meaningful to calculate $\sigma_{g_{1}}$ . The third and fourth terms in Eq. (10) decrease with *N*_tot_, whereas the first and second terms remain constant. Therefore, as *N*_tot_ increases, $\sigma_{g_{1}}$ approaches the sum of the first two terms. Consistent with this, Fig. 7(c) shows that $\sigma_{g_{1}}$ approaches a plateau for *N*_tot_ greater than ∼10^6^ photons per second. We used a minimisation algorithm (*fminsearch* in MATLAB) to find the value of *T*_bin_ that minimises $\sigma_{g_{1}}$ for various values of *N*_tot_. Figure 7(c) shows the optimal *T*_bin_ as a function of *N*_tot_, along with the corresponding $\sigma_{g_{1}}$ values, for three values of $g_{1}$ (1, 0.36, and 0). The optimal *T*_bin_ has a minor dependence on the value of $g_{1}$ (i.e., *τ* value).

Here, we analysed which part of the $g_{1}$ curve provides the highest contrast (Δ*g*_1_) and contrast-to-noise ratio $\left(\text{CNR}=\Delta g_{1}/\sigma_{g_{1}}\right)$ for changes in *αD*_b_ at different depths. We computed $g_{1}$ curves for both the baseline and a 10% increase in *αD*_b_ within a 3 mm thick layer starting at depths of 0, 9, or 18 mm below the surface, as shown in Fig. 1. Figure 8 shows Δ*g*_1_ and CNR for CW-DCS (0 to 10 ns time gate) (Fig. 8(a)), and for the late gate (2 to 3 ns) (Fig. 8(b)). The corresponding values of $\sigma_{g_{1}}$ used to calculate CNR are shown in Fig. 8(c).

The late time gate shows a much higher contrast for the deeper-located Δ*αD*_b_ (18 mm depth) compared to CW-DCS, as expected. Note, the CNR axis scale in Fig. 8(b) is 10 times smaller than in Fig. 8(a). Therefore, although the late gate has a higher contrast, its CNR is comparable to that of CW-DCS because it contains significantly fewer photons, as shown in Fig. 8(c). The smallest $\sigma_{g_{1}}$ (at *g*_1_ = 0) differs from the largest $\sigma_{g_{1}}$ (at *g*_1_ = 1) by a factor of $\sqrt{2}$ , consistent with the derivation in the Appendix. Figure 8 shows that the optimal values of $g_{1}$ are around 0.35, where Δ*g*_1_ and CNR are highest, which is consistent with the e^-1^ value recommended in [26].

If photons with all pathlengths are used (CW-DCS), the shape of the Δ*g*_1_ curve varies with the depth at which Δ*αD*_b_ occurs (Fig. 8(a)), indicating depth-dependent contrast. However, this dependence on depth is minor, as the peak of Δ*g*_1_ shifts only from *g*_1_ = 0.34 to *g*_1_ = 0.42 when the contrast moves from 0 to 9 mm depth.

Here, we compared different 0.5 ns time gates for assessing changes in *αD*_b_ (Δ*αD*_b_) at various depths. We used the same three-layered model as for Fig. 8, where *αD*_b_ was increased by 10% in a 3 mm layer at various depths. We computed $g_{1}$ curves using 0.5 ns time gates, starting at 0 ns and incremented by 0.5 ns across the time axis. For each gate, we calculated Δ*g*_1_ curve as in Fig. 8(a, b) and found its maximum value. Figure 9 shows the maximum Δ*g*_1_, the corresponding $\sigma_{g_{1}}$ for the baseline $g_{1}$ , and the corresponding CNR for different 0.5 ns time gates.

The early gates show higher Δ*g*_1_ for Δ*αD*_b_ in shallower layers, whereas the later gates show higher Δ*g*_1_ for Δ*αD*_b_ in deeper layers, as expected and shown in Fig. 9(a). For Δ*αD*_b_ at 27 mm depth, the earliest time gate shows Δ*g*_1_ ≈ 0, confirming that early photons are not sensitive to changes in deep layers [48]. For Δ*αD*_b_ at depths greater than 9 mm, Δ*g*_1_ increases at later time gates. However, the number of photons decreases, and hence $\sigma_{g_{1}}$ increases, at later time gates, as shown in Fig. 9(b). Figure 9(c) shows the maximum CNR for different time gates, which relies on the results in Fig. 9(a) and in Fig. 9(b). As Δ*αD*_b_ occur at greater depths, the optimal time gate shifts to later times. The results confirm that TD-DCS has the capability to recover Δ*αD*_b_ at multiple depths.

## 4. Discussion

We quantitatively analysed the accuracy of using modelling solutions in NIRS, which provide the distribution of times of flight of photons (DTOFs), for modelling $g_{1}$ in DCS. The benefit of using existing solutions in time-domain NIRS, as opposed to deriving solutions in time-domain DCS, is that the effect of the instrument response function (IRF) can be easily included. In MC simulations, the effect of the IRF can be incorporated by adding a random number to the photon arrival time, where the random number’s probability distribution function is defined by the IRF [49]. For analytical solutions of the DE, the IRF can be included by convolving the DTOF with the IRF [24]. The recently developed MC code, which simulates TD-DCS measurements from first principles, provides an alternative approach for modelling $g_{1}$ [49]. We applied the same zero-boundary condition, which neglects internal reflections, across all modelling approaches. The relative accuracy of modelling approaches, and the results of this study, are independent of the specific boundary condition used. For modelling measured signals, the extrapolated boundary condition is recommended [23], as implemented in the MATLAB codes we used [40].

We observed a consistent discrepancy between *μ*′_s_*L* and *Y* for photon arrival times less than ∼1 ns. *Y* is consistently smaller than *μ*′_s_*L* (Fig. 2). Consequently, the recovered α*D*_b_ is underestimated when *μ*′_s_*L* is used instead of *Y*, which aligns with the theoretical expectations [17]. Different modelling approaches produce similar $g_{1}$ curves, with discrepancies when early photons or short *ρ* are used (Fig. 4). These discrepancies diminish for later time gates (after ∼1 ns) and longer *ρ* (more than ∼20 mm) (Fig. 6). Early photons can be excluded by using later time gates in TD-DCS or by using a longer *ρ* in CW-DCS, as shown in Fig. 3(a). For a given time gate in TD-DCS, *ρ* has no influence on the values of the $g_{1}$ curve (Fig. 4(c)).

Our results in Figs. 4 to 6 are consistent with those in [50], where the authors investigated errors in recovered *αD*_b_ at short *ρ*. The authors found a good fit between $g_{1}$ calculated using MC (the first approach in this study) and using an analytical solution of the CDE for a homogeneous medium (the fourth approach in this study), but at the cost of errors in the recovered *αD*_b_ values. The authors used a scaling factor for accurate recovery of *αD_b_* [50]. This is consistent with the analysis in Figs. 2, 3, and 6, where the results are independent of the baseline *αD*_b_. The errors in the recovered *αD*_b_ observed in this study (up to ∼9%, but typically a few percent) (Fig. 6) are consistent with those reported in [51], where the error is below 12% for *ρ* > 0.2 cm, but smaller than those reported in [50], where the error is about 20% ± 10% for *ρ* = 5 mm. The effects of *μ*_a_ and *μ*′_s_, as shown in Figs. 5 and 6, are consistent with previous findings [50,51]. In particular, *μ*_a_ has a much smaller effect on $g_{1}$ as compared to *μ*′_s_ [52].

We substituted the expression for $g_{1}$ into the equation for its standard deviation $\left(\sigma_{g_{1}}\right)$ , expressing $\sigma_{g_{1}}$ as a function of $g_{1}$ rather than the decay rate. The standard deviation of an exponentially decaying autocorrelation function, such as $g_{1}$ in DCS, was derived by Koppel [28] and applied to DCS by Zhou et al. [26,27]. For a medium with both static and dynamic particles, the decay rate of $g_{1}$ might be better modelled with multiple decay constants [53]. Although the proposed version of the equation for $\sigma_{g_{1}}$ does not use the decay rate, it is a version of the equation for $\sigma_{g_{1}}$ that assumes the autocorrelation function has a single exponential decay rate. Instead of using the decay rate, the proposed equation uses the value of $g_{1}$ at *τ* = *T*_bin_. Wohland et al. [29] demonstrated that the theoretical estimations of $\sigma_{g_{1}}$ based on Koppel’s derivation does not accurately predict the measured $\sigma_{g_{2}}$ . A more advanced theoretical framework for statistical analysis of the standard deviation and bias in FCS, including derivations, were presented by Saffarian et al. [43], which can be applied in DCS.

Analysis of noise (Fig. 7) showed that optimising *T*_bin_ of a correlator can significantly reduce $\sigma_{g_{1}}$ . We found that the optimal *T*_bin_ is approximately the inverse of the count rate *N*_tot_, which is when the average number of photons detected per *T*_bin_, i.e., 〈*n*〉, is ∼1 (Fig. 7(c)). The value of $\sigma_{g_{1}}$ approaches a plateau for count rates much higher than ∼10^5^ photons per second (Fig. 7(c)), and the choice of *T*_bin_ is less critical at higher count rates (Fig. 7(a)). Although the noise equation is an approximation and relies on multiple assumptions [28,29], it shows a strong dependence on *T*_bin_ and that the optimal *T*_bin_ is inversely related to the count rate. Count rates measured in DCS are typically on the order of 10^5^ photons s^-1^, for which the optimal *T*_bin_ is approximately 10^−5^ s. However, the decay of $g_{1}$ usually begins at *τ* ∼ 10^−6^ s (Fig. 4). Since *τ* cannot be smaller than *T*_bin_, the optimal *T*_bin_ should be the smallest of: approximately the inverse of *N*_tot_ and the value of *τ*. A multi-tau correlator arrangement uses a similar approach, where calculations at longer *τ* use longer *T*_bin_, which reduces noise and computational time [26]. Furthermore, the results imply that different *T*_bin_ values should be used across different time gates depending on the number of photons detected within these time gates.

A limitation of the noise model is that it does not account for correlations between noise at different *τ* values. TD-DCS allows calculation of the $g_{1}$ curve at thousands of *τ* values, with the smallest possible spacing equal to *T*_bin_. To model the $g_{1}$ curve with noise, a random Gaussian noise (with zero mean and scaled by $\sigma_{g_{1}}$ ) is added independently at each *τ*. With this approach, calculating $g_{1}$ for an additional *τ* value is equivalent to taking a new measurement of $g_{1}$ . A phantom study is needed to establish the correlation of noise at different *τ* values for the $g_{2}$ (or $g_{1}$ ) curve. $\sigma_{g_{2}}$ can be calculated using measured intensity traces, as carried out for fluorescence correlation spectroscopy by Wohland et al. [29]. In addition to theoretical noise, measured data will also encompass all sources of instrumental noise in DCS, which is important to address, as the laser and detector have been shown to strongly influence the overall performance of TD-DCS [54].

For optimal parameter recovery, $g_{1}$ (or $g_{2}$ ) values can be weighted by their standard deviation. However, this will have little effect as the maximum and minimum values of $\sigma_{g_{1}}$ (at *g*_1_ = 1 and *g*_1_ = 0, respectively) differ by no more than $\sqrt{2}$ , as shown in Fig. 8(c) and derived in the Appendix.

We have analysed the theoretical $g_{1}$ curves, but expect the findings to also apply to the measured $g_{2}$ curves, with additional effects due to the IRF and laser coherence length. Samaei et al. [54] compared the performance of different laser sources and found that the IRF has a greater impact on the overall performance than the temporal coherence. The impact of the IRF [55] and the laser coherence length [56] can be incorporated in the data analysis used in this study. Future research can focus on developing practical guidelines for analysing data measured with TD-DCS systems.

## 5. Conclusions

We compared four established approaches for modelling $g_{1}$ in TD-DCS. The analysis included a comparison of pathlengths (*μ*′_s_*L*) and momentum transfers (*Y*) obtained from MC simulations, as well as their probability distributions *P*(*μ*′_s_*L*) and *P*(*Y*), for the anisotropy factor *g* = 0.9 and 0. We found that *μ*′_s_*L* is consistently greater than *Y*, particularly for early photons (up to ∼0.3 ns). This discrepancy leads to an underestimation of the recovered flow parameter *αD*_b_ by up to 9% (typically a few percent) when *μ*′_s_*L* is used instead of *Y*. In contrast, the analytical solution of the DE showed only up to 2% error. *Y* approaches *μ*′_s_*L* for late photons and longer *ρ*, allowing modelling solutions in NIRS to be directly applied to $g_{1}$ in DCS with small discrepancies.

We proposed a more convenient version of the equation for the standard deviation of $g_{1}$ (or $g_{2}$ ) as a function of $g_{1}$ (or $g_{2}$ ), which does not require the knowledge of the decay rate. The standard deviation of $g_{2}$ can be significantly reduced in data processing by increasing the correlator time bin width (*T*_bin_). We found that the lowest standard deviation occurs if the photon count rate is higher than the inverse of *T*_bin_, which can be varied across different time gates due to different count rates. However, at short τ (e.g., 10^−6^ s), if a photon count rate is not high enough (i.e., less than 10^6^ photons s^-1^), *T*_bin_ may be set to *τ* since *T*_bin_ cannot be longer than *τ*.

## Figures and Tables

**Fig. 10.**
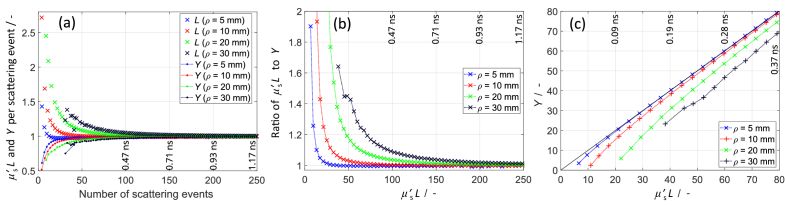
Same analysis as in Fig. 2, but for isotropic scattering (*g* = 0) and the same *μ*′_s_ = 1 mm^−1^. (a) Mean *μ*′_s_*L* and *Y* per scattering event after different number of scattering events. (b) Ratio of *μ*′_s_*L* to *Y* at different photon arrival times $\left(t_{\text{a}}\right)$ , where $t_{\text{a}}$ is converted to *L* using *L* = *ct_a_*. (c) Direct comparison of *μ*′_s_*L* and *Y*. The corresponding $t_{\text{a}}$ values (units ns) are indicated in each panel.

**Fig. 11.**
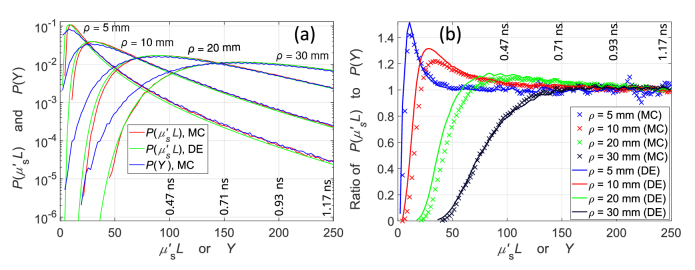
Same analysis as in Fig. 3, but for isotropic scattering (*g* = 0) and the same *μ*′_s_ = 1 mm^−1^. (a) *P*(*μ*′_s_*L*) and *P*(*Y*) calculated using *L* and *Y* from MC simulations or an analytical solution of the DE. (b) Ratio of *P*(*μ*′_s_*L*) to *P*(*Y*). The x-axis represents *μ*′_s_*L* for *P*(*μ*′_s_*L*) and *Y* for *P*(*Y*).

## Data Availability

Monte Carlo data and MATLAB scripts used in this study were made available under the terms of the Creative Commons Attribution 4.0 License [57].

## Appendix

In Section 3.1 (Figs. 2 and 3), we presented a comparison between *μ*′_s_*L* and *Y*, along with their probability distributions *P*(*μ*′_s_*L*) and *P*(*Y*), for forward scattering (*g* = 0.9 and *μ*_s_ = 10 mm^-1^). To investigate the generality of the relation between *μ*′_s_*L* and *Y*, we repeated the comparison for isotropic scattering (*g* = 0 and *μ*_s_ = 1 mm^-1^). The number of scattering events decreases by a factor of 10, and the values of *μ*′_s_*L* and *Y* per scattering event increase by a factor of 10, as expected and shown in Fig. 10(a). Since there are fewer scattering events, the early photons have a greater chance of reaching a detector with much smaller than average scattering angles (∼0.5 *Y* per scattering event) and much longer than average scattering steps (∼2.5 *L* per scattering event), as shown in Fig. 10(a). Therefore, the ratio of *μ*′_s_*L* to *Y* is higher for isotropic scattering (Fig. 10(b)) than forward scattering (Fig. 2(b)).

*Y* starts from lower values (Fig. 10(c)), causing *P*(*Y*) to shift to the left as shown in Fig. 11(a). *P*(*μ*′_s_*L*) calculated using Monte Carlo (MC) simulations and the DE solution align more closely when g = 0 (Fig. 11) than when g = 0.9 (Fig. 3), and especially for larger *ρ*, as expected since the DE always assumes g = 0.

Similar as in [26,27], we briefly show the derivation of the noise equations (Eqs. (9) and 10) for the autocorrelation functions $g_{1}$ and $g_{2}$ . Most of the parameters were defined in Section 2.2. Koppel [28] considered an exponentially decaying correlation function f(τ) :

(11)
$$f(\tau )=\beta {\text{e}}^{-\Gamma \tau }$$

where Γ is the exponential decay rate. Koppel derived an equation for the variance of such a function [28]. After normalising by the factor ${\left\langle n\right\rangle }^{4}$ , where 〈*n*〉 is the average number of photons detected during time *T*_bin_, the equation for the variance of f(τ) is:

(12)
$$\begin{array}{l} \sigma_{\text{f}}{\left(mT_{\text{bin}}\right)}^{2}=\frac{\beta^{2}}{N_{\text{bin}}}\frac{(1+e^{-2\Gamma T_{\text{bin}}})(1+e^{-2\Gamma mT_{\text{bin}}})+2m(1-e^{-2\Gamma T_{\text{bin}}})e^{-2\Gamma mT_{\text{bin}}}}{\left(1-e^{-2\Gamma T_{\text{bin}}}\right)}\\ \;+\frac{2\beta }{N_{\text{bin}}\langle n\rangle }(1+e^{-2\Gamma mT_{\text{bin}}})+\frac{1}{N_{\text{bin}}{\left\langle n\right\rangle }^{2}}(1+\beta e^{-\Gamma mT_{\text{bin}}}) \end{array}$$

where *N*_bin_ is the number of time bins $(N_{\text{bin}}=T_{\text{meas}}/T_{\text{bin}})$ , *m* is the index of the time delay $(m=\tau /T_{\text{bin}})$ , and 〈*n*〉 is the average number of photons within time *T*_bin_, $\left\langle n\rangle =N_{\text{tot}}/(T_{\text{meas}}/T_{\text{bin}}\right)$ . Following the approach by Wohland et al. [29], we substituted Eq. (11) into Eq. (12), resulting in the following equation that does not include the Γ parameter:

(13)
$$\begin{array}{l} \sigma_{\text{f}}{\left(mT_{\text{bin}}\right)}^{2}=\frac{(\beta^{2}+f{\left(T_{\text{bin}}\right)}^{2})(\beta^{2}+f{\left(mT_{\text{bin}}\right)}^{2})+2m(\beta^{2}-f{\left(T_{\text{bin}}\right)}^{2})f{\left(mT_{\text{bin}}\right)}^{2}}{N_{\text{bin}}(\beta^{2}-f{\left(T_{\text{bin}}\right)}^{2})}\\ \;+\frac{2\beta }{N_{\text{bin}}\langle n\rangle }(1+f{\left(mT_{\text{bin}}\right)}^{2}/\beta^{2})+\frac{1}{N_{\text{bin}}{\left\langle n\right\rangle }^{2}}(1+f(mT_{\text{bin}})) \end{array}$$

Kopell [28] defined the signal, i.e. the correlation function f(τ) , as the correlation level above the accidental correlation background, where the accidental correlation background is the value of f(τ) at τ→∞ . The value of $g_{1}$ decays from 1 to 0, whereas the value of $g_{2}$ decays from 1+β to 1 based on the Siegert relation, $g_{2}(\tau )=1+\beta g_{1}{\left(\tau \right)}^{2}$ [17]. Therefore, we derive the variance of the function $g_{2}(\tau )-1$ , which decays to 0. The variance of $g_{2}$ is the same as that of $g_{2}(\tau )-1$ , since subtracting a constant from a variable does not change its variance. To derive the variance of $g_{2}$ , we substitute $g_{2}(\tau )-1$ for f(τ) into Eq. (13), as well as the definitions of *N*_bin_, *m*, and 〈*n*〉 in the previous paragraph. The resulting equation for the variance of $g_{2}$ in terms of $g_{2}$ is Eq. (9) presented in Section 2.2.

At *τ* = 0, the value of $g_{1}$ is 1, hence the variance of $g_{1}$ is given by (Eq. (10)):

(14)
$$\sigma_{g_{1}}{\left(0\right)}^{2}=\frac{2\;T_{\text{bin}}}{T_{\text{meas}}}\frac{\left(1+g_{1}{\left(T_{\text{bin}}\right)}^{2}\right)}{1-g_{1}{\left(T_{\text{bin}}\right)}^{2}}+\frac{4}{N_{\text{tot}}}+\frac{2\;T_{\text{meas}}}{T_{\text{bin}}}\frac{1}{{N_{\text{tot}}}^{2}}$$

For very long *τ*, the value of $g_{1}$ approaches 0, hence the variance of $g_{1}$ will approach:

(15)
$$\underset{\tau \to \infty }{\lim}(\sigma_{g_{1}}{\left(\tau \right)}^{2})=\frac{T_{\text{bin}}}{T_{\text{meas}}}\frac{\left(1+g_{1}{\left(T_{\text{bin}}\right)}^{2}\right)}{1-g_{1}{\left(T_{\text{bin}}\right)}^{2}}+\frac{2}{N_{\text{tot}}}+\frac{T_{\text{meas}}}{T_{\text{bin}}}\frac{1}{{N_{\text{tot}}}^{2}}$$

The largest $\sigma_{g_{1}}$ (at *τ* = 0, Eq. (14)) and the smallest $\sigma_{g_{1}}$ (at τ→∞ , Eq. (15)) differ by a factor of $\sqrt{2}$ , as also shown in Fig. 8(c):

(16)
$$\sigma_{g_{1}}{\left(\tau =0\right)}^{2}=2\underset{\tau \to \infty }{\lim}(\sigma_{g_{1}}{\left(\tau \right)}^{2})$$

The same is true for the difference between $g_{2}(0)$ and $g_{2}(\tau \to \infty )$ :

(17)
$$\sigma_{g_{2}}{\left(\tau =0\right)}^{2}=2\underset{\tau \to \infty }{\lim}(\sigma_{g_{2}}{\left(\tau \right)}^{2})$$
